# A retrospective comparison of double-hooked locking plates versus non-locking plates in minimally invasive percutaneous plate osteosynthesis for the treatment of comminuted distal fibular fractures accompanied by tibial Pilon fractures

**DOI:** 10.1186/s13018-023-03731-7

**Published:** 2023-04-10

**Authors:** Jun-Hong Liu, Qiang Zhang, Guo-Hua Wei, Liang Liu, Xin Mu, Mao-Lin Li, Zong-De Wu

**Affiliations:** Foot and Ankle Department, Sichuan Orthopaedic Hospital, Chengdu, 610000 Sichuan China

**Keywords:** Pilon fracture, Comminuted distal fibula fractures, Double-hooked locking plate, Minimally invasive percutaneous plate osteosynthesis (MIPPO), Efficacy analysis

## Abstract

**Background:**

Surgical approach and fixation material are crucial in the treatment of comminuted distal fibular fractures accompanied by tibial Pilon fractures. This study compared the efficacy of double-hooked locking plates and anatomic plates in minimally invasive percutaneous plate osteosynthesis (MIPPO) for the treatment of comminuted distal fibular fractures accompanied by tibial Pilon fractures.

**Methods:**

Clinical data were collected from 96 patients diagnosed with comminuted distal fibular fractures accompanied by tibial Pilon fractures who had undergone MIPPO. Patients in the study group (*n* = 48) received double-hooked locking plate fixations and the control group (*n* = 48) received anatomical plate fixations. The operating time, intraoperative bleeding, length of hospital stays, full weight-bearing time, fracture healing time and complication rates in the two groups were compared. The quality of fracture reduction was evaluated using the Burwell–Chamley imaging scoring system; the ankle function was assessed based on the American Orthopaedic Foot and Ankle Society Ankle-Hindfoot Score.

**Results:**

Patients in the study group had shorter operating time, less bleeding, significantly shorter hospital stays, and shorter time to full weight-bearing as well as fracture healing compared to the control group (*P* < 0.05). Additionally, the post-operative complication rates were significantly lower in the study group (6.16% vs. 22.92%) (*P* < 0.05), but there was no significant difference in the fracture reduction rate between the two groups (*P* > 0.05). Patients in the study group experienced better ankle recovery than those in the control group (93.75% vs. 75.00%) (*P* < 0.05).

**Conclusion:**

Double-hooked locking plates have advantages in the treatment of comminuted distal fibular fractures accompanied by tibial Pilon fractures during MIPPO due to their shorter operating time and less intraoperative bleeding, as well as shorter hospital stays, full weight-bearing time and fracture healing time, fewer complications and better ankle recovery. Therefore, double-hooked locking plates are worthy of clinical application.

## Introduction

Minimally invasive percutaneous plate osteosynthesis (MIPPO) was first proposed by Krettek et al. [1]. During MIPPO, an incision of approximately 3 cm in length was made at one end of the fracture, and then a steel plate was inserted percutaneously; after the force line and alignment of the fracture were restored, the screw was inserted percutaneously for fixation. Generally, MIPPO does not require cutting the fracture end, so it can maximize the preservation of blood supply to the fracture and is conducive to fracture healing [2, 3]. Some scholars believe that MIPPO is suitable for treating comminuted fractures with lots of bone fragments, numerous cancellous bones and a large contact surface [4]. At present, MIPPO is widely applied in the treatment of Pilon fractures with fibular fractures and has shown promising therapeutic results [5, 6].

Pilon fractures are fractures of the distal tibial joint and the weight-bearing portion of the ankle and are usually caused by high-energy injuries accompanied with direct axial impact [7]. Given that they are frequently caused by falls from great heights and automobile accidents, Pilon fractures are uncommon in clinical practice (accounting for just 1–10% of tibial fractures) [8]. Pilon fractures usually involve the metaphysis and may be accompanied with fibular fractures. Fibular fractures complicate 90% of Pilon fractures, and these fractures typically involve comminuted distal fibular fractures, which makes the condition more severe [9, 10].

In recent years, it has been pointed out that most of the comminuted fractures of distal fibula were caused by high-energy impact of pronation abduction. Moreover, high non-union rate of comminuted distal fibular fractures requires internal fixation for complete reduction [11, 12]. To achieve a complete reduction in the comminuted fibula fractures, it is necessary to calibrate the length and rotation angle of the fibula. Clinically, common bone screws cannot fix the comminuted fractures of distal fibula, so it is imperative to discover a suitable treatment to cope with the difficult reduction. Anatomical plate is a common material for fixation of fibular fractures. In terms of its mechanism of action, the fracture site is compressed and fixed by the friction force generated when the bone surface is in close contact with anatomical plate so that the bone morphology is stably attached [13]. The double-hooked locking plate is a special deformation at the distal end of the locking plate. Owing to the double-hooked shape, the distal fibula fracture fragment can be pulled back for reduction. Moreover, the combination of the double-hooked locking plate and locking screws allows a firmer fixing of fibula fragments. Currently, double-hooked locking plates have been applied in external ankle fractures and achieved good clinical effect [14, 15]. In this study, our goal was to retrospectively review the clinical data of 96 patients with Pilon fractures combined with comminuted distal fibular fractures who were treated with double-hooked locking plates or anatomic plates under MIPPO. Through this retrospective analysis, we intend to provide a reference for the clinical treatment of comminuted distal fibular fractures that are accompanied by tibial Pilon fractures.

## Materials and methods

### Research subjects

A retrospective analysis was conducted on clinical data of patients with Pilon fractures combined with comminuted distal fibular fractures admitted to Sichuan Orthopaedic Hospital from January 2019 to December 2021. A total of 96 patients were included in the study based on the inclusion and exclusion criteria. According to the plate used to fix the fibula, the patients were assigned to control group (received anatomical plate fixation, *n* = 48) or study group (received double-hook locking plate fixation, *n* = 48). The study was approved by the Ethics Committee of Sichuan Orthopaedic Hospital (Ethics No: KKY-2021-023-01).

Inclusion criteria: patients with (1) computerized tomography (CT) and X-ray results of comminuted distal fibular fractures accompanied by tibial Pilon fractures; (2) closed, fresh fractures with an interval of ≤ 2 weeks between fracture and surgery; (3) indications for surgery.

Exclusion criteria: patients with (1) severe organ dysfunctions or cardiovascular diseases; (2) open fractures, or old/pathological fractures; or (3) coagulation disorders.

### Surgical manipulation of the fibula fixation

**Fig. 1:**
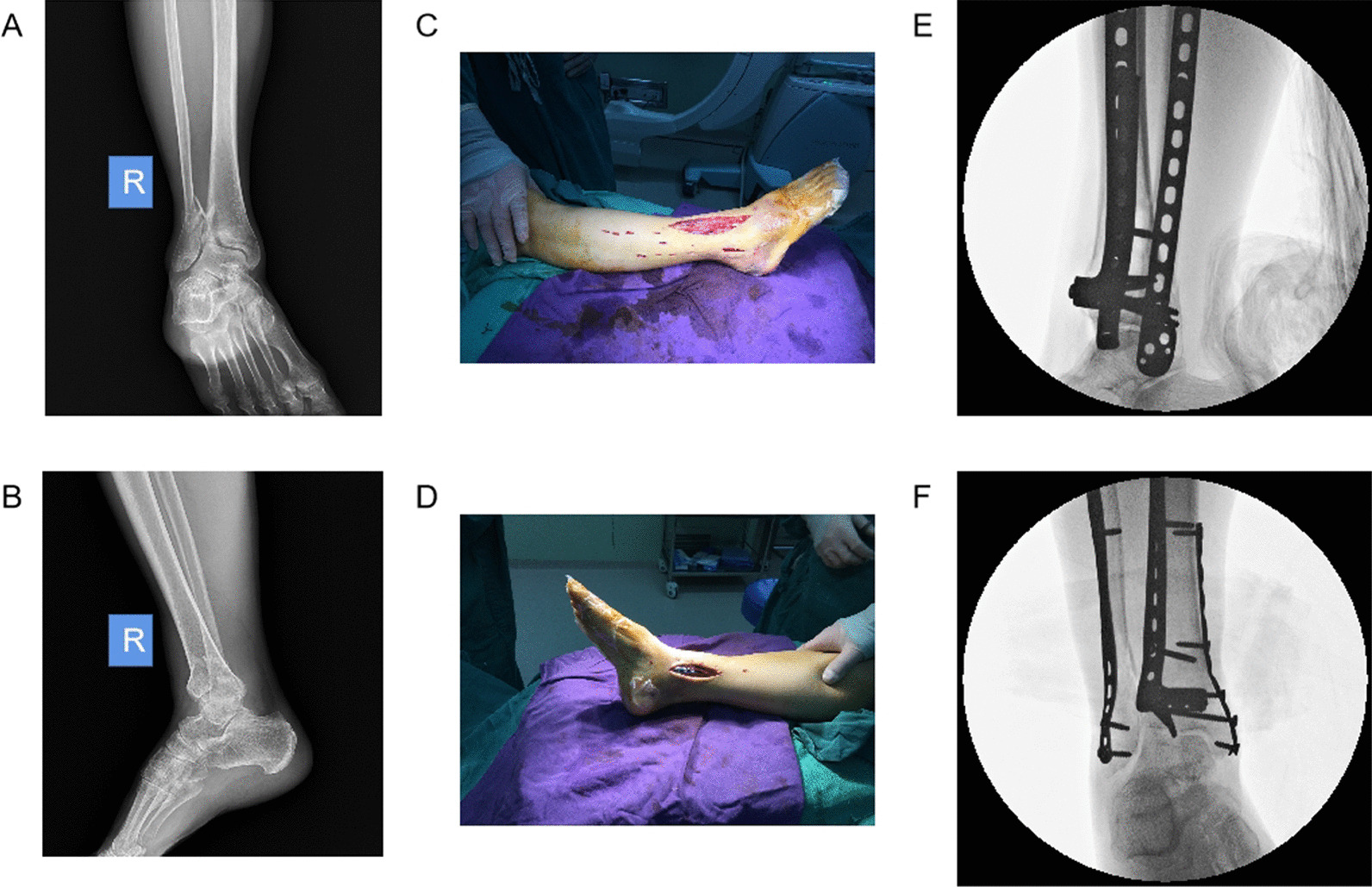
Performance of a patient in the control group before and during fracture fixation. **A** Original anteroposterior radiograph of fracture for the patient. **B** Original lateral radiograph of fracture for the patient. **C** Surgical incision of the right leg of the patient (external). **D** Surgical incision of the right leg of the patient (internal). **E** Anteroposterior radiograph for intraoperative fluoroscopy of the patient. **F** Lateral radiograph for intraoperative fluoroscopy of the patient

Control group: The patients were placed in supine position, and then underwent nerve block anaesthesia + intubation for general anaesthesia followed by the tying of tourniquets to their injured limbs. Fixation of the fibula before or after repositioning and fixation of the Pilon fractures were performed based on the preoperative repositioning design. Later, the distal fibula was exposed after a 2–3 cm incision was made using MIPPO. The anatomical plate (Zimmer, USA) was inserted retrogradely through the subcutaneous tunnel at the distal end. The position of the plate was then adjusted, and 3–4 self-tapping screws were placed to fix the plate at the distal fibula. Next, the distal fibula was moderately tracted using a soft tissue envelope and manual reduction, with the plate positioned proximal to the midline of the fibula. Finally, 2–3 self-tapping screws were placed percutaneously to fix the fibula fractures (Fig. 1).

**Fig. 2:**
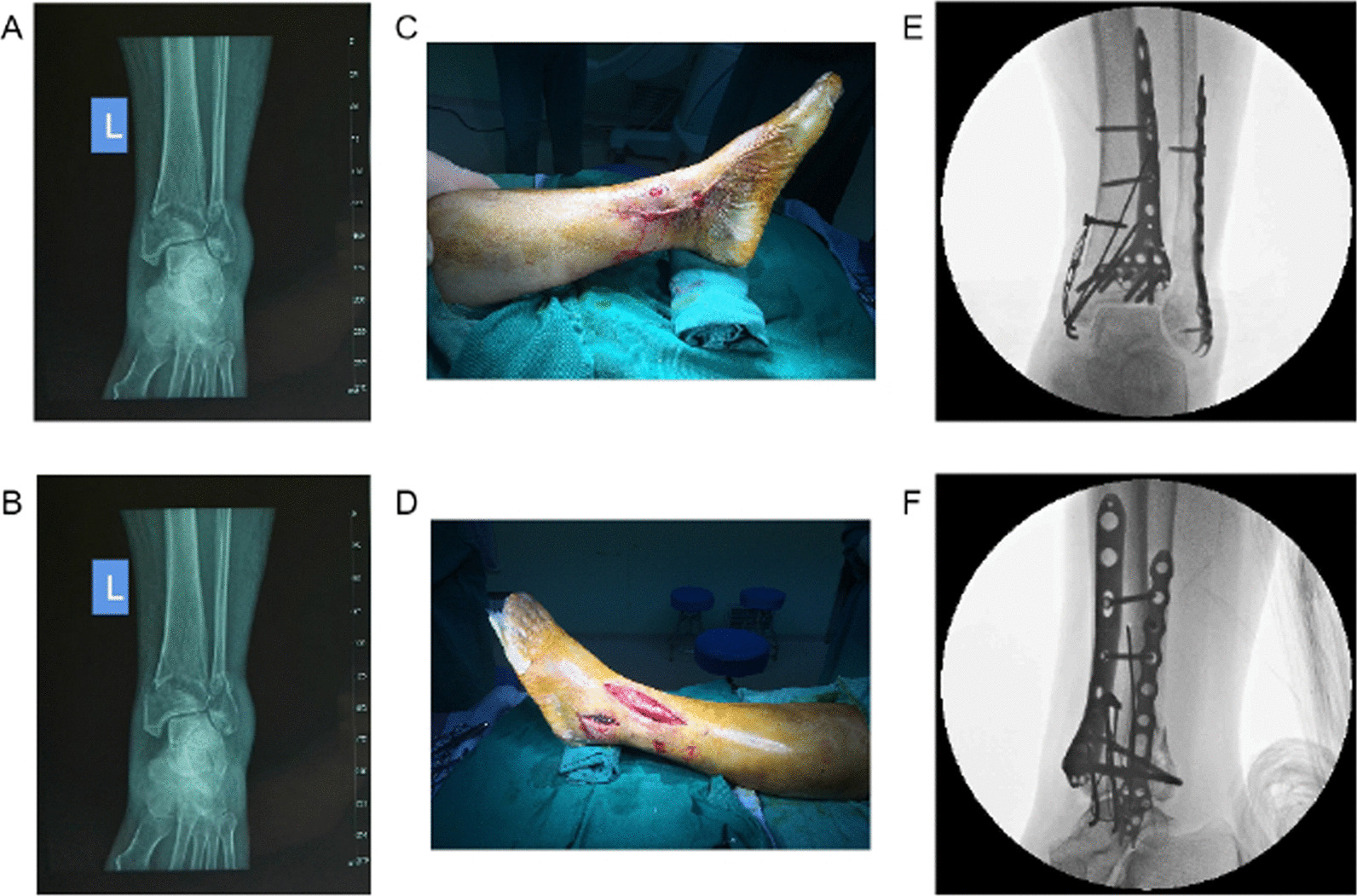
Performance of a patient in the study group before and during fracture fixation. **A** Original anteroposterior radiograph of fracture for the patient. **B** Original lateral radiograph of fracture for the patient. **C** Surgical incision of the left leg of the patient (internal). **D** Surgical incision of the left leg of the patient (external). **E** Anteroposterior radiograph for intraoperative fluoroscopy of the patient. **F** Lateral radiograph for intraoperative fluoroscopy of the patient

Study group: The patients were placed in supine position and underwent nerve block anaesthesia + intubation for general anaesthesia, and tourniquets were tied to the injured limbs. Likewise, fixation of the fibula before or after repositioning and fixation of the Pilon fractures were carried out depending on the preoperative repositioning design. Subsequently, MIPPO was adopted to make a 2–3 cm incision in the distal fibula, and the innominate tubercle of the distal fibula (i.e. near the insertion of the anterior talofibular ligament at the distal fibula) was exposed. The double-hooked locking plate (Aplus, China) was inserted retrogradely, and later the plate position was adjusted. After that the distal double grasping hooks tightly hooked both sides of the innominate tubercle, and 3–5 locking screws were placed to fix the distal fibula. The distal fibula was tracted using the soft tissue envelope and manual reduction, and the plate was positioned proximal to the midline of the fibula. Finally, 3 locking screws were placed proximally to fix the fibula fractures (Fig. 2).

### Observed indicators

General data: Age, gender, body mass index (BMI), causes (including car accident, fall from height, and others), and AO Foundation/Orthopaedic Trauma Association (AO/OTA) Fracture Classification [16] of patients in the study and control groups were collected and recorded.

Perioperative-related indicators: Perioperative-related indicators of the two groups were compared, including operating time, intraoperative bleeding, length of hospital stays, full weight-bearing time, and fracture healing time.

Post-operative complication rate: The delayed healing, malunion, incision infection, broken or loose internal fixation, traumatic arthritis and skin margin necrosis of patients in the two groups were recorded, and the complication rate was counted after the operation.

Fracture reduction: The quality of the fracture reduction was collected from both groups of patients and were evaluated according to Burwell–Chamley imaging scoring system [17], including anatomical reduction, general reduction and reset error. The fracture reduction rate of the two groups was calculated as fracture reduction rate = (anatomical reduction + general reduction)/total number of cases × 100%.

Ankle function scores at the final follow-up: Ankle function scores were collected from both groups of patients. The ankle function was scored following the American Orthopaedic Foot and Ankle Society (AOFAS) Ankle-Hindfoot Score [18], and four items are included in the scoring system: pain, quality of daily life, joint mobility and joint stability. The total score is 100, of which 90 ~ 100 is the best, 75 ~ 89 is good, 50 ~ 74 is acceptable, and < 50 is poor.

### Statistical analysis

SPSS20.0 statistical software was used to process the data. Enumeration data were expressed as *N* (%), and the χ^2^ test was used for comparison between groups; measurement data were displayed as mean ± standard deviation, paired t test was used for intra-group comparison, and independent t test was adopted for inter-group comparison. *P* < 0.05 is considered a statistically significant difference.

## Results

### Analysis of general data of patients in both groups

As shown in Table 1, a total of 96 patients were included in this study. The 48 patients (23 men and 25 women) in the control group had a mean age of 51.21 ± 4.35 years. The 48 patients (20 men and 28 women) in the study group had a mean age of 49.83 ± 4.68 years. Based on the AO/OTA Fracture Classification, there were 15 cases of C1, 14 cases of C2 and 19 cases of C3 in the study group, and 15 cases of C1, 13 cases of C2, and 20 cases of C3 in the control group. When comparing the age, gender, BMI, causes and AO/OTA Classification of the two groups, no statistically significant difference was found (*P* > 0.05).

**Table 1:** General data of patients in both groups

	Control group (*n* = 48)	Study group (*n* = 48)	*χ*^2^*/t*	*P*
Age (years)	51.21 ± 4.35	49.83 ± 4.68	1.487	0.140
Gender (m/f)	23/25	20/28	0.276	0.600
BMI (kg/m^2^)	24.45 ± 1.16	24.30 ± 1.36	0.600	0.550
Causes			0.527	0.768
Car accident	18	16		
Fall from height	26	26		
Others	4	6		
AO/OTA Classification			0.063	0.969
C1	15	15		
C2	13	14		
C3	20	19		

Measurement data were expressed as mean ± standard deviation; enumeration data were expressed as *N*

### Perioperative related indicators of patients in both groups

As shown in Table 2, patients in the study group showed shorter operating time and less intraoperative bleeding than the control group, with a statistically significant difference (*P* < 0.05). In post-operative observation, the study group presented significantly shorter than the control group in terms of length of hospital stays, full weight-bearing time and fracture healing time (*P* < 0.05).

**Table 2:** Relevant indicators of both groups during hospitalization

	Control group (*n* = 48)	Study group (*n* = 48)	*t*	*P*
Operating time (min)	119.27 ± 30.59	103.85 ± 37.36	2.212	0.029
Intraoperative bleeding (ml)	104.27 ± 30.59	87.88 ± 24.25	2.910	0.005
Length of hospital stays (d)	19.52 ± 2.69	17.34 ± 3.29	3.543	0.001
Full weight-bearing time (weeks)	19.69 ± 2.01	16.85 ± 2.03	6.836	< 0.001
Fracture healing time (weeks)	16.77 ± 2.07	14.28 ± 2.68	5.083	< 0.001

Measurement data were expressed as mean ± standard deviation

### Complication rates of patients in both groups

Table 3 displays that there was one case of delayed healing, one case of superficial incision infection, and one case of broken or loose internal fixation in the study group, with an overall complication rate of 6.25%. In the control group, there were two cases of delayed healing, two ca Double-hooked ses of malunion, two cases of superficial incision infection, two cases of broken or loose internal fixation, two cases of traumatic arthritis, and one case of skin margin necrosis, with a total complication rate of 22.92%. Overall, the complication rate in the study group was lower than that in the control group, and the difference was statistically significant (*P* < 0.05).

**Table 3:** Complications of patients in both groups

Complications	Control group (*n* = 48)	Study group (*n* = 48)	*χ*^2^	*P*
Delayed healing	2 (4.17)	1 (2.08)		
Malunion	2 (4.17)	0 (0.00)		
Superficial incision infection	2 (4.17)	1 (2.08)		
Broken or loose internal fixation	2 (4.17)	1 (2.08)		
Traumatic arthritis	2 (4.17)	0 (0.00)		
Skin margin necrosis	1 (2.08)	0 (0.00)		
Total incidence	11 (22.92)	3 (6.25)	3.872	0.049

Enumeration data were expressed as *N* (%)

### Fibula fracture reduction quality of patients in both groups

**Fig. 3:**
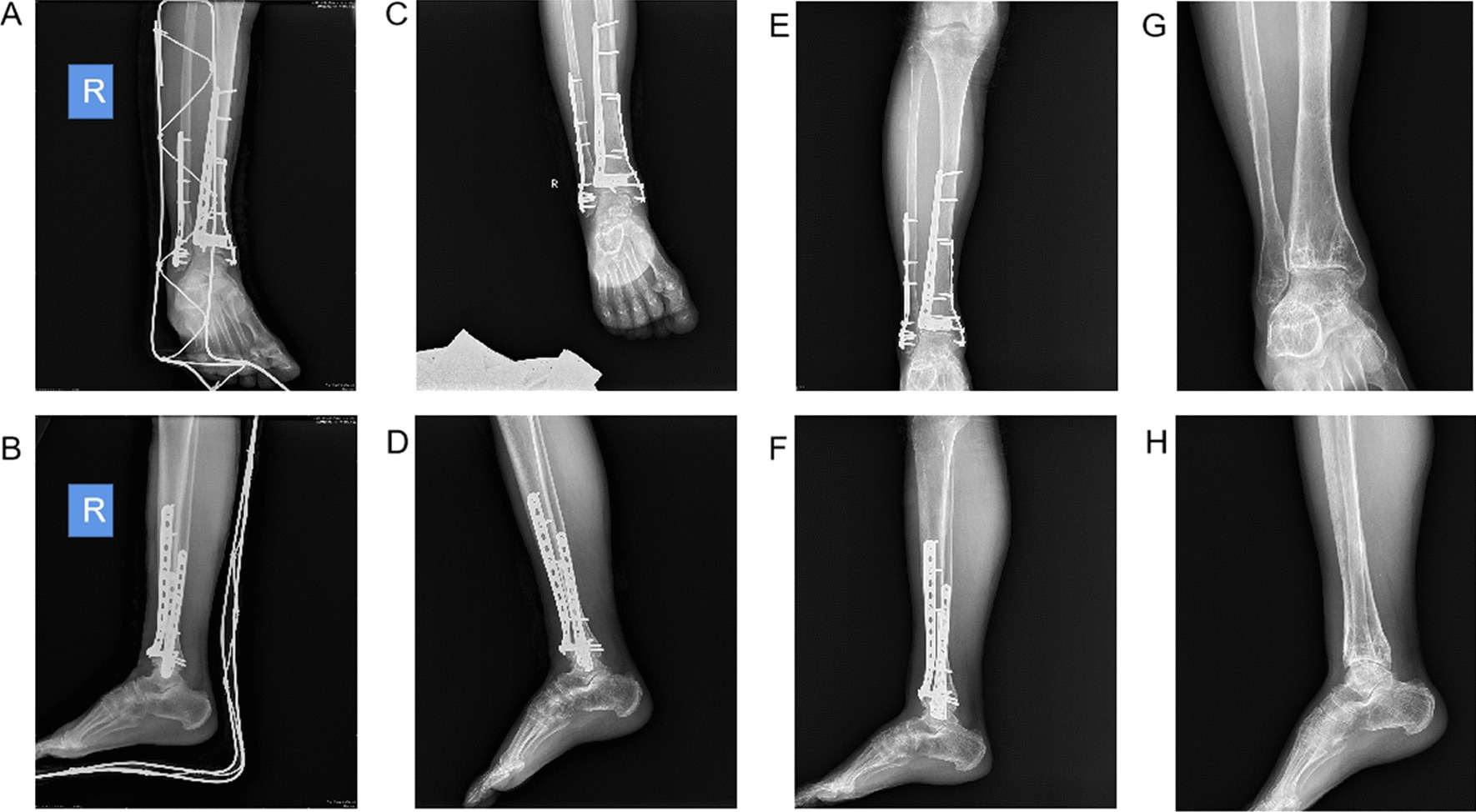
Performances of one patient in the control group receiving fracture fixation for 3 days, 1 month, 1 year and removing internal fixation after 2 years. **A** Anteroposterior radiograph 3 days after surgery. **B** Lateral radiograph 3 days after surgery. **C** Anteroposterior radiograph 1 month after surgery. **D** Lateral radiograph 1 month after surgery. **E** Anteroposterior radiograph 1 year after surgery. **F** Lateral radiograph 1 year after surgery. **G** Anteroposterior radiograph for removing internal fixation after 2 years. **H** Lateral radiograph for removing internal fixation after 2 years. *Note* the patient was the same as the one in Fig. 1

**Fig. 4:**
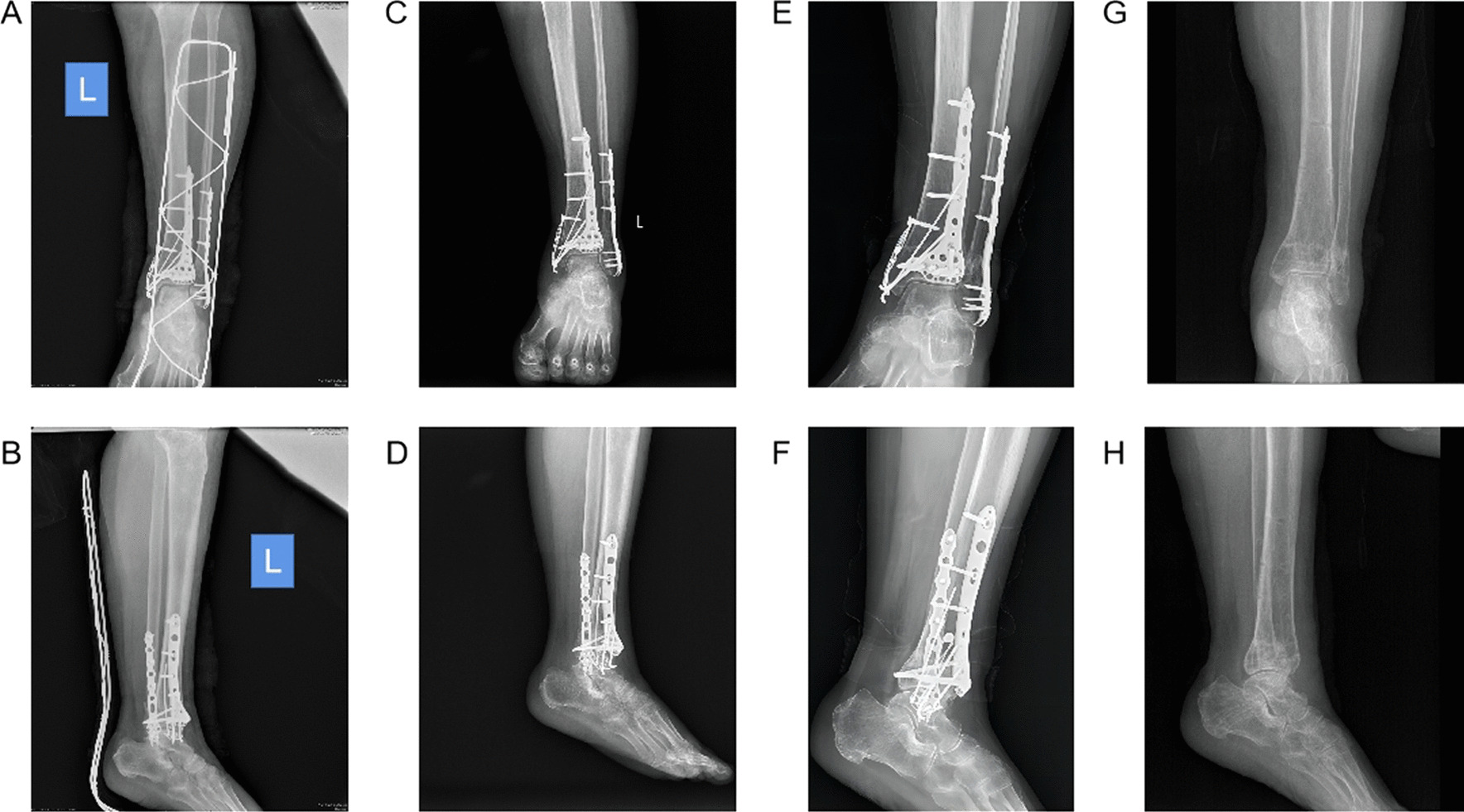
Performances of one patient in the study group receiving fracture fixation for 3 days, 1 month, 1 year and removing internal fixation after 2 years. **A** Anteroposterior radiograph 3 days after surgery. **B** Lateral radiograph 3 days after surgery. **C** Anteroposterior radiograph 1 month after surgery. **D** Lateral radiograph 1 month after surgery. **E** Anteroposterior radiograph 1 year after surgery. **F** Lateral radiograph 1 year after surgery. **G** Anteroposterior radiograph for removing internal fixation after 2 years. **H** Lateral radiograph for removing internal fixation after 2 years. *Note* the patient was the same as the one in Fig. 2

The fibula fracture reduction in the two groups of patients is shown in Table 4. In the study group, 42 patients had anatomical reduction (87.50%), four patients had general reduction (8.33%) and one patient had reset error (2.08%), giving a fracture reduction rate of 95.83%. As for the control group, 34 patients had anatomical reduction (70.83%), 11 patients had general reduction (22.92%) and three patients had reset errors (6.25%), giving a fracture reduction rate of 93.75%. However, no statistically significant difference was identified (*P* > 0.05) in the fibula fracture reduction rates between the two groups (Figs. 3 & 4).

**Table 4:** Fracture reduction quality of patients in both groups

Reduction quality	Control group (*n* = 48)	Study group (*n* = 48)	*χ*^2^	*P*
Anatomical reduction	34 (70.83)	42 (87.50)	0.211	0.646
General reduction	11 (22.92)	4 (8.33)		
Reset error	3 (6.25)	1 (2.08)		
Fracture reduction rate	45 (93.75)	46 (95.83)		

Enumeration data were expressed as *N* (%)

### Recovery of the ankle joint of patients in both groups

The recovery of the ankle joint in both groups was assessed using the Burwell–Chamley imaging scoring system and the results are shown in Table 5. In the study group, 26 patients showed excellent recovery, 19 patients showed good recovery, three patients showed acceptable recovery, and no patients showed poor recovery; the overall excellent rate was 93.75%. For the control group, 14 patients had excellent recovery, 22 patients had good recovery, seven patients had acceptable recovery, and five patients had poor recovery; the overall excellent rate was 75.00%. Accordingly, the study group showed better ankle joint recovery than the control group (*P* < 0.05).

**Table 5:** Recovery of the ankle joint of patients in both groups

	Excellent	Good	Acceptable	Poor	Excellent rate (%)	*χ*^2^	*P*
Control group (*n* = 48)	14	22	7	5	75.00	6.544	0.011
Study group (*n* = 48)	26	19	3	0	93.75		

Enumeration data were expressed as *N*

## Discussion

Nowadays, most studies on Pilon fractures combined with comminuted distal fibular fractures focus more on the Pilon fractures but less on fixation of fibula fractures. Related research has stated that stabilization of fibular fractures plays an important role in reducing distal tibial malunion and post-traumatic ankle arthropathy [19]. The main focus of this study was on the efficacy of a double-hooked locking plate or anatomical plate under MIPPO, aiming to provide data support for the clinical treatment of patients with comminuted distal fibular fractures accompanied by tibial Pilon fractures.

In this study, patients who received double-hooked locking plate fixation displayed less intraoperative bleeding as well as shorter operating time, full weight-bearing time, and fracture healing time than those who received anatomical plate treatment. In regard to the design of the double-hooked locking plate, the part at the upper end of the plate is gradually narrowed into an arrow-like shape, which aids in sliding the plate to fix the fibula during MIPPO; moreover, the shape of the screw is also relatively narrow. When compared to traditional anatomical plates, such design of the double-hooked locking plate can reduce the stimulation of soft tissues and faster healing of tissues. The incision caused by MIPPO is small so that the inserting of the plate may destroy the integrity of the subcutaneous tissue. Fortunately, the design of the double-hooked locking platemakes it possible to lessen the anatomical peeling injury of subcutaneous tissue, lower intraoperative blood loss, and shorten the time of tissue healing. Our results also supported the aforementioned findings.

The post-operative complications of patients were also a focus of this investigation. Briefly speaking, only one superficial incision infection and one delayed healing occurred in patients treated with double-hooked locking plates, with a probability of delayed healing of 2.08% and no malunion; whereas patients treated with anatomical plates developed delayed healing, malunion, superficial incision infection, broken or loose internal fixation, traumatic arthritis and skin margin necrosis. Based on a series of designs, the locking plate system locks the screw to the plate to prevent the screws and plates from slipping, thereby forming a single cross-beam structure with a fixed angle [20]. Such structure converts the shear forces of internal fixation under load into pressure between the screw and the bone, which is more conducive to fracture fixation. Also, it can reduce loose internal fixation, maintain the blood supply to the bone surface, and speed up faster recovery of the fracture and limb functions. The anatomical plate, otherwise, mainly presses the bone mostly by friction at the bone-plate junction; however, the close adhesion of the plate to the bone surface may result in poor blood supply to the bone surface, which would slow fracture healing and recovery [20]. Double-hooked locking plate fixation of comminuted fibula fractures not only results in fewer problems than anatomical plate, but it also offers superior therapeutic efficacy and is more suited to patient post-operative rehabilitation.

In terms of reduction in the comminuted distal fibular fractures, double-hooked locking plates are superior to anatomical plates. In the study group, patients were treated by double-hooked locking plates. More specifically, the two grappling hooks of the plate were hooked at the innominate tubercle of the distal fibula; the two ends of the comminuted fibula fractures were bridged and fixed with the help of effective anatomical landmarks of the distal fibula; the original anatomical form of the fibula could then be reconstructed through the anatomical form of the plate; notably, the bridging and fixation by MIPPO technology through the double-hooked locking plates did not occur fibula rotation and angulation. The two grappling hooks at the distal fibula plus the plate proximal to fibula finally constitute a multiplanar stereoscopic fixation construct. Additionally, the fixing points of the two grappling hooks were positioned at the distal fibula and were vertical to the horizontal plane, and the external screw fixing points of the plate were vertical to the sagittal plane. By comparison, the double-hooked locking plates presented a multiplanar fixing effect while traditional anatomical plates didn’t, they are superior to the traditional ones in controlling rotation and angulation. The Aplus double-hooked locking plate selected in this study is an anatomical shape plate for fibula based on Asian race data, while the traditional ZIMMER anatomical plate is designed based on European and American race data. As it stands, the double-hooked locking plates adopted in this study are more suitable for the Chinese situation in terms of anatomical performance.

As a retrospective analysis, this study has certain limitations. For example, the low incidence of tibial Pilon fractures associated with comminuted distal fibular fractures resulted in a small sample size for this study. Thus, randomised controlled trials with multi-centres and large sample sizes should be carried out in the future to further verify the results obtained in this study.

## Conclusion

In the treatment of patients with tibial Pilon fractures associated with comminuted distal fibular fractures, the advantages of double-hooked locking plates are not only reflected in shorter operating time and less intraoperative bleeding, but also in terms of shorter hospital stays, full weight-bearing time and fracture healing time, fewer complications, and better ankle recovery. Simply put, double-hooked locking plate may be an appropriate therapeutic strategy for MIPPO treating comminuted distal fibular fractures accompanied by tibial Pilon fractures, and it is worthy of application in clinical practice.

## Data Availability

The datasets used and/or analysed during the current study are available from the corresponding author upon reasonable request.

## Abbreviations

MIPPO: Minimally invasive percutaneous plate osteosynthesis
AOFAS: American Orthopaedic Foot and Ankle Society
ORIF: Open reduction and internal fixation
